# Simultaneous Determination of Nine Active Compounds of the Traditional Chinese Medicinal Prescription Shaoyao-Gancao-Tang and Analysis of the Relationship between Therapeutical Effect and Compatibility of Medicines

**DOI:** 10.1155/2014/521038

**Published:** 2014-11-06

**Authors:** Guangwei Zhu, Guijun Zhang, Meng Wang, Jingjuan Wang, Weixin Zeng, Xiaomei Gao

**Affiliations:** ^1^School of Traditional Chinese Materia Medica, Beijing University of Traditional Chinese Medicine, Beijing 100102, China; ^2^Department of Pharmacy, Beijing Shijitan Hospital, Beijing 100038, China

## Abstract

A simple and sensitive HPLC-DAD detection method was established for the simultaneous determination of nine compounds including oxypaeoniflorin, albiflorin, paeoniflorin, benzoylpaeoniflorin, glycyrrhizic acid, liquiritin, isoliquiritin, liquiritigenin, and isoliquiritigenin in the Traditional Chinese Medicinal Prescription Shaoyao-Gancao-Tang (SGT) and we analyze the relationship between therapeutical effect and compatibility of medicines by using an Agilent extend-C18 column at a flow rate of 1 mL/min. The column temperature was maintained at 30°C and the detection wavelength was set at 230 nm for oxypaeoniflorin, albiflorin, paeoniflorin, benzoylpaeoniflorin, and glycyrrhizic acid; 276 nm for liquiritin and liquiritigenin; 360 nm for isoliquiritin and isoliquiritigenin. The total contents of the nine compounds in SGT varied from 4.65 to 20.06 mg/mL. The results of this study showed that the content of chemical compounds of Traditional Chinese Medicinal Prescription is mainly influenced by the dosage and compatibility of medicines and the therapeutical effect of Traditional Chinese Medicinal prescription is mainly influenced by the dosage and compatibility of medicines. The method could be suitable for quality control of SGT with bioactive multicompounds.

## 1. Introduction

SGT is a Traditional Chinese Prescription containing Radix Paeoniae alba (stir-baked with vinegar) and Radix Glycyrrhizae (stir-baked with honey) with the ratio of 1 : 1 and is commonly used to relieve spasm and pain. It was originally described in Treatise on Febrile Diseases, a medical classic written by Zhongjing Zhang in the 3rd century.

**Figure 1:**
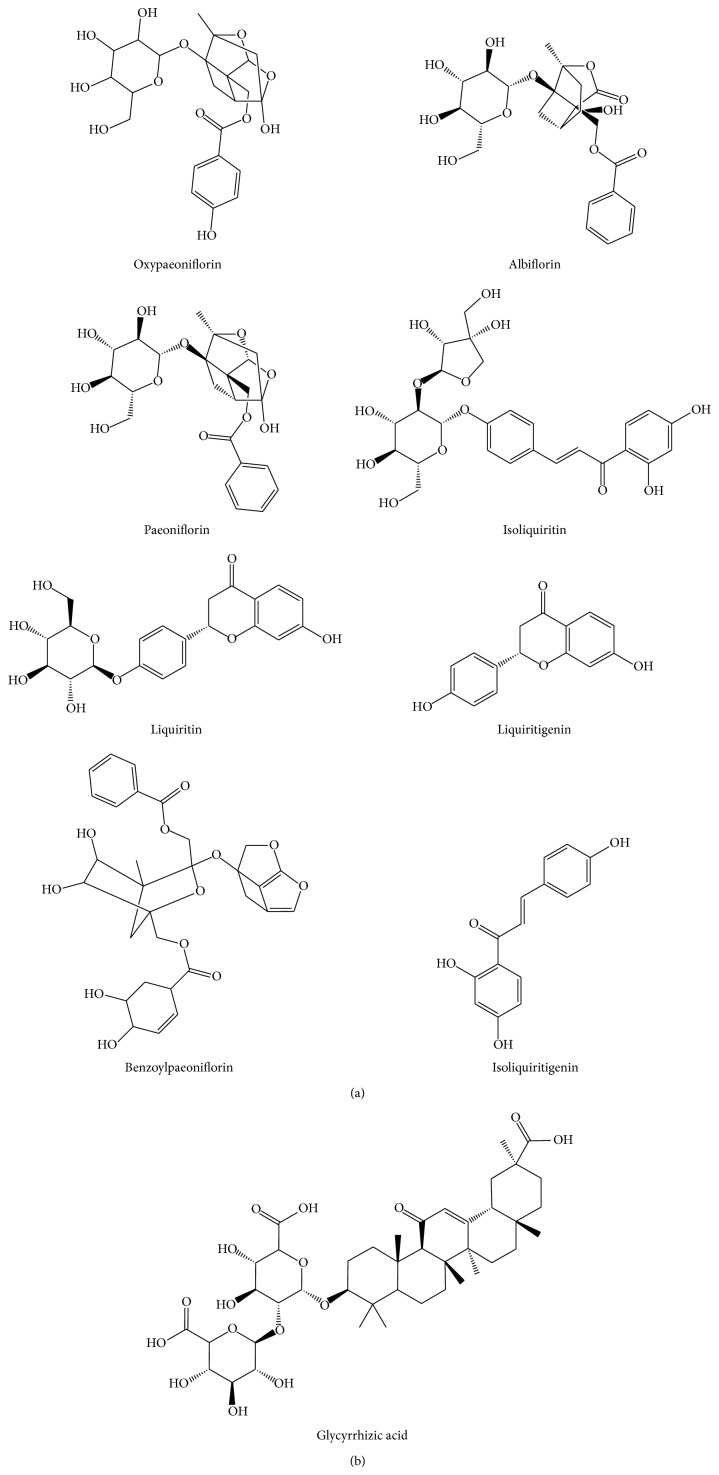
The structures of nine constituents in SGT.

SGT contains many chemical constituents. Oxypaeoniflorin, albiflorin, paeoniflorin, liquiritin, isoliquiritin, liquiritigenin, benzoylpaeoniflorin, isoliquiritigenin, and glycyrrhizic acid (Figure 1) are the most important bioactive constituents reported at present. Albiflorin has the effects in the prevention of osteoporosis and inhibitory effects on DNA cleavage [1, 2]. Paeoniflorin has been widely investigated as an anti-inflammatory, cognitive enhancer, endothelium-dependent vasodilator, and neuroprotective agent [3–7]. Liquiritigenin and glycyrrhizic acid inhibit liver cell injury and were given intravenously for the treatment of chronic viral hepatitis and cirrhosis in Japan [8, 9]. It has also proven itself effective in the treatment of autoimmune hepatitis in one clinical trial [10]. These effects are related to the inhibition of cortisol metabolism within the kidney [11]. Other side effects include [12] headache, transient visual loss, tachycardia, cardiac arrest, hypokalaemia, acute kidney, muscle weakness, myopathy, myoglobinuria, rhabdomyolysis, and increased body weight. Isoliquiritigenin is a chalcone compound and has valuable pharmacological properties such as antioxidant, anti-inflammatory, anticancer, and antiallergic activities. Isoliquiritin and liquiritin have the pharmacological activities of antiulcer [13], antioxidative [14], antimicrobial [15], and antitumor [16] activities and others. Oxypaeoniflorin and benzoylpaeoniflorin have the activities of anti-inflammatory, antianxiety, analgesia, spasmolysis, and anticonvulsant [17]. When the nine compounds are mixed in different proportions, they also have different effects. Traditionally, SGT is used clinically for its antispasmodic and muscle relaxation effects in the treatment of leg cramps, stomachache, and menstrual colic pain [18–21]. In the later days, SGT changed, and also its clinical efficacy changed (Table 1).

Modern research has shown that SGT has the remarkable curative effect to the many kinds of diseases. Recently, other pharmacological properties of SGT, such as anti-inflammatory, antioxidative, and neuroprotective effects, have also been reported [22, 23]. In Japan, SGT is one of the predominant traditional Chinese formulations clinically used and is widely applied for the treatment of abdominal pain [24], sometimes together with analgesics and antispasmodics such as anticholinergic drug [25]. As SGT is also used to promote the healing of peptic ulcer, it may also combine with synthetic antisecretory drugs as a histamine H_2_-receptor antagonist and triple therapy using OAM, a combination of a proton pump inhibitor to reduce gastric acid secretion and two antibiotics to remove* Helicobacter pylori* [26].

**Figure 2:**
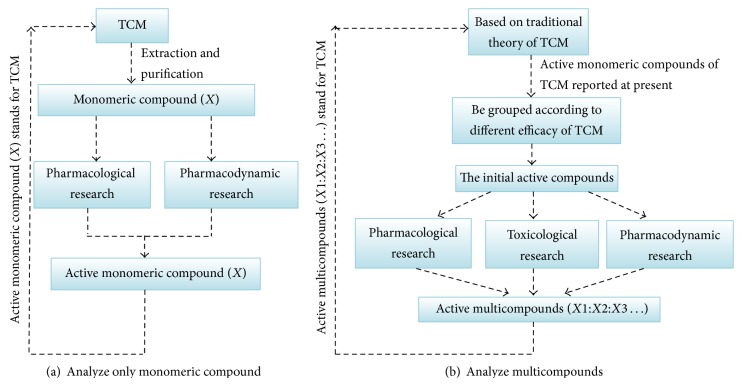
Studies on TCM.

However, very few reports on the quality control of SGT have been published so far. Most methods for quality control of TCM only analyze monomeric compound, which is insufficient as they do not reveal all the compounds present in the chromatographic profile [27]. These studies are not in accord with Traditional Chinese Medical (TCM) theory. The theory suggests that the efficacy of TCM is decided by many different kinds of chemical constituents with bioactivities (Figure 2). Basically, these chemicals are similar to TCM in terms of treatment effect.

Studies have shown that a high percentage of relevant studies on traditional Chinese medicine are in Chinese databases. Fifty percent of systematic reviews on TCM did not search Chinese databases, which could lead to a bias in the results [28]. Many systematic reviews of TCM interventions published in Chinese journals are incomplete, some contained errors or were misleading [29]. In this study, we focused on establishing an effective method to evaluate the quality of SGT for its safe use in clinical practice. In the present study, a HPLC-DAD method was first developed to analyze multicompounds including oxypaeoniflorin, albiflorin, paeoniflorin, liquiritin, isoliquiritin, liquiritigenin, benzoylpaeoniflorin, isoliquiritigenin, and glycyrrhizic acid for quality control of SGT.

Figure 2 illustrates two research methods of traditional Chinese Medicine. The former focus on monomeric compound and the latter focus on multicompounds. TCM is a complex system, which consists of many kinds of herbs. Each herb performs one or more specific functions when used alone. However, they will play a role in the treatment of more when used in combination. Why? The answer is that TCM contains many chemical components, they influence each other (sometimes produce one or more new substances) and result in different therapeutical effects. These effects were caused not by a monomeric compound, but by multicompounds. So, the later method should be the best choice.

## 2. Materials and Methods 

### 2.1. Chemicals and Reagents

The reference compounds of oxypaeoniflorin, albiflorin, paeoniflorin, liquiritin, isoliquiritin, liquiritigenin, benzoylpaeoniflorin, isoliquiritigenin, and glycyrrhizic acid (purity ≥ 98%) were purchased from Chengdu Pufeide Biotech Co., Ltd. of China (Chengdu, China). Medicinal materials were purchased from Hebei anguo Chinese Herbal Medicine Co., Ltd. of China (Hebei, China). Acetic acid was purchased from Tianjin Chemical Regent Co., Ltd. (Tianjin, China), methanol and acetonitrile were HPLC-grade (Fisher, USA) and high purity water was obtained from Wahaha Co., Ltd. (Hangzhou, China).

### 2.2. Instrumentation and Separation Conditions

Liquid chromatographic analysis was performed on an Agilent HPLC-DAD system, which was equipped with an Agilent 1260 infinity DAD (DEAA306741), an Agilent 1260 infinity autosampler (DEAAC23211), an Agilent 1260 infinity column heater (DEACN25004), an Agilent 1260 infinity pump (DEAB709020), and an Agilent extend-C18 (4.6 mm × 250 mm, 5 *μ*m) column. The binary gradient elution system consisted of solvent A (acetonitrile) and solvent B (0.1% acetic acid water). Optimum separation was achieved by using the gradient program described in Table 2. The column temperature was maintained at 30°C. The autosampler was conditioned at 25°C and injection volume was 10 *μ*L. The flow rate was 1 mL/min. The raw data was detected by an HPLC-DAD (Agilent1260, USA) and the wavelengths were shown in Table 3.

### 2.3. Sample Preparation

Medicinal materials (Table 4) were immersed in distilled water (1 : 10, w/v) and boiled twice for 30 min at each time. The filtrates from each decoction were mixed and concentrated to 1/2 of its original volume; the concentrate solution was thoroughly mixed and then centrifuged again at 8000 rpm for 20 min at 4°C. Then filter the concentrate solution with a 0.45 *μ*m filter. For HPLC analysis, the solution with the volume of 1 mL was dissolved in methanol at a final volume of 10 mL. Before injection, it was filtered through a 0.22 *μ*m filter.

### 2.4. Preparation of Standard Solution

The appropriate amount of oxypaeoniflorin, albiflorin, paeoniflorin, liquiritin, isoliquiritin, liquiritigenin, benzoylpaeoniflorin, isoliquiritigenin, and glycyrrhizic acid were separately weighed and dissolved together in methanol to achieve a standard working solution of nine different concentrations; the concentrations of the nine reference compounds were 42 *μ*g/mL, 70.2 *μ*g/mL, 190.8 *μ*g/mL, 102 *μ*g/mL, 41.2 *μ*g/mL, 44.8 *μ*g/mL, 47.2 *μ*g/mL, 4.12 *μ*g/mL, and 440 *μ*g/mL. The calibration curves were constructed by analyzing the mixed solution, and the series of working solutions within the ranges of 0.84~8.4 *μ*g/mL for oxypaeoniflorin, 1.4~14 *μ*g/mL for albiflorin, 3.82~38.2 *μ*g/mL for paeoniflorin, 2.04~20.4 *μ*g/mL for liquiritin, 0.08~0.8 *μ*g/mL for isoliquiritin, 0.9~9.0 *μ*g/mL for liquiritigenin, 0.94~9.4 *μ*g/mL for benzoylpaeoniflorin, and 8.8~88 *μ*g/mL for glycyrrhizic acid, respectively. All solutions were prepared in dark brown calibrated flasks and stored at 4°C.

### 2.5. Method Validation

The HPLC method was validated in terms of linearity, precision, stability, repeatability, and recovery. The validation was performed based on the relative peak areas (RPAs). Linear regression analysis was employed to construct calibration curves. The data was expressed as mean ± standard deviation. And relative standard deviation (RSD) was used to evaluate precision, stability, repeatability, and recovery.

## 3. Results

### 3.1. Chromatography

**Figure 3:**
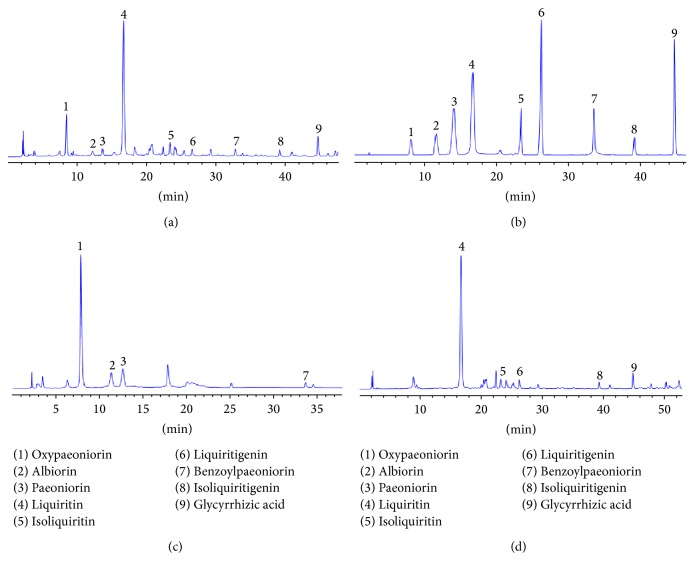
HPLC chromatograms of SGT test sample (a), standard mixture (b), test sample without Radix Glycyrrhizae (stir-baked with honey) (c), and test sample without Radix Paeoniae alba (Stir-baked with vinegar) (d).

The chromatogram of test sample and mixed standard compounds were shown in Figure 3. In chromatograms (a), (b), (c), and (d), the nine peaks marked with 1–9 are oxypaeoniflorin, albiflorin, paeoniflorin, liquiritin, isoliquiritin, liquiritigenin, benzoylpaeoniflorin, isoliquiritigenin, and glycyrrhizic acid. The retention time is 8.514 min, 12.195 min, 13.599 min, 16.525 min, 23.957 min, 26.116 min, 33.600 min, 40.931 min, and 44.705 min, respectively. In chromatograms (a), (c), and (d), the unknown peaks are probably licorice and ketone, licorice alcohol, isoliquiritigenin alcohol, methyl gallate, ethyl gallate, galloyl benzoic acid, and formononetin which was based on published references. Further research will be performed soon in this area in order to provide sufficient evidence for the identification of those unknown peaks.

### 3.2. Calibration Curves

Linearity was evaluated by analyzing six injection quantities of standard solutions, and then the calibration curves were constructed by plotting the peak areas and the injection quantity of 2 *μ*L, 5 *μ*L, 10 *μ*L, 15 *μ*L, and 20 *μ*L of each compound. Linear regression equation, linear range, limit of detections (LODs), and limit of quantitation (LOQs) for nine standard substances are given in Table 5.

### 3.3. Precision, Stability, Repeatability, and Recovery

The precision was performed by six replicate determinations of the standard working solution. Stability was evaluated by analyzing the solutions stored at room temperature (about 25°C) at different time points (0, 4, 8, 12, and 24 h after preparation). The solutions for stability test included mixed solutions of reference standard and SGT sample solutions. Five replicates were performed for the test. These data confirmed that the nine compounds were stable within 24 h at 25°C and their RSD values were between 0.53% and 2.50%. The repeatability was examined by six replications of a sample. In the recovery test, samples were prepared at three concentration levels in triplicate by spiking known quantities of each of the nine standards into the SGT sample and then extracted and analyzed according to the described procedures. The validation data are shown in Table 6.

### 3.4. Contents of Nine Compounds in SGT

**Figure 4:**
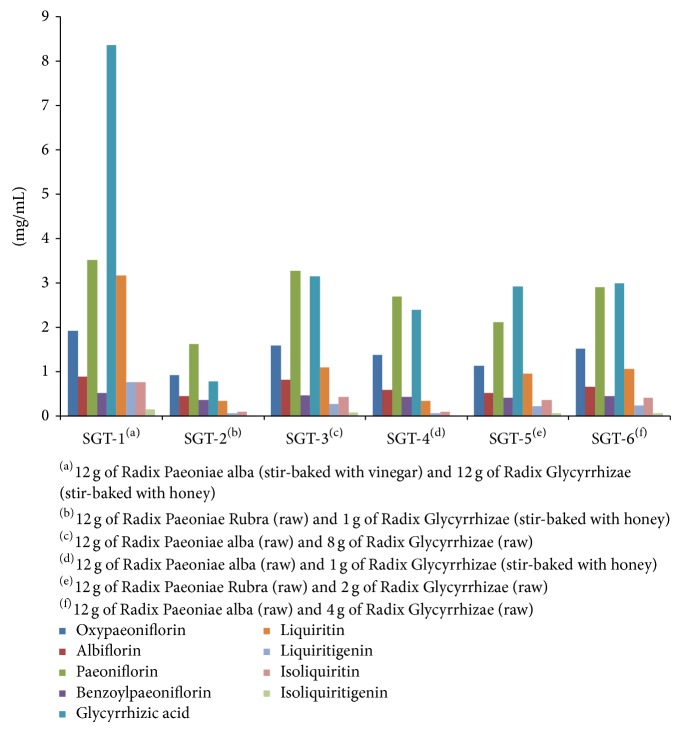
The contents of nine compounds in SGT (mg/mL) (*n* = 3).

The HPLC data demonstrated that oxypaeoniflorin, albiflorin, paeoniflorin, benzoylpaeoniflorin, glycyrrhizic acid, liquiritin, isoliquiritin, liquiritigenin, and isoliquiritigenin are present in the extract of SGT. The present study proves that different compatibility of medicines has different contents. The content of nine compounds in SGT is shown in Table 7 and Figure 4.

## 4. Discussion

### 4.1. Optimization of HPLC Conditions

The combination of acetonitrile and 0.1% aqueous phosphoric acid (v/v) proved to be an optimal mobile phase system for the purpose of chromatographic separation compared with acetonitrile and 0.02% aqueous phosphoric acid (v/v), acetonitrile and 0.05% aqueous phosphoric acid (v/v). The data showed that separation of peak 2 and peak 3 is not very good in the condition of acetonitrile and 0.02% aqueous phosphoric acid (v/v), acetonitrile and 0.05% aqueous phosphoric acid (v/v). The resolution is about 1.00. The column temperature was maintained at 30°C by comparing with 35°C, 40°C. The data showed that separation of peak 1 and peak 5 is not very good at 35°C and 40°C. The resolutions were about 1.01 and 1.03, and the retention times were 7.04 min and 20.57 min which were shorter than the normal value. The flow rate of 1 mL/min was determined by testing different flow rates which were 0.5 mL/min, 0.8 mL/min, and 1.5 mL/min. Final gradient elution method was chosen by testing different gradient elution methods.

### 4.2. Selections of Nine Active Compounds

The theory of Traditional Chinese Medicine holds that the therapeutical effect of Traditional Chinese Medicinal Prescription is due to multicompounds. Oxypaeoniflorin, albiflorin, paeoniflorin, and benzoylpaeoniflorin are the major active compounds of Radix Paeoniae alba. Liquiritin, isoliquiritin, liquiritigenin, isoliquiritigenin, and glycyrrhizic acid are the major active compounds of Radix Glycyrrhizae [30]. The nine compounds all have antispasmodic and analgesic effect. As a result, the nine compounds of SGT should be considered for study.

### 4.3. Correlation between Content, Dosage, and Compatibility of Medicines

The content of chemical compounds of Traditional Chinese Medicinal Prescription is mainly influenced by the dosage and compatibility of medicines.

The dosage of herbs remains constant while the compatibility of herb medicines is changed; different compatibility of herb medicines can lead to different content of compounds. Take SGT-2 and SGT-5 for example: SGT-2 is composed of Radix Paeoniae Rubra (12 g, raw) and Radix Glycyrrhizae (1 g, stir-baked with honey), and SGT-5 is composed of Radix Paeoniae alba (12 g, raw) and Radix Glycyrrhizae (1 g, stir-baked with honey); the two drugs have the same dosage but different herbs. Therefore, the contents of chemical compounds are different. The contents are (mg/mL) 0.92, 0.45, 1.62, 0.37, 0.78, 0.34, 0.06, 0.10, and 0.01 and 1.14, 0.52, 2.12, 0.41, 2.92, 0.97, 0.22, 0.36, and 0.06, respectively.

The compatibility of herb medicines remains constant while the dosage level is changed, different dosage of herb medicines can lead to different content of compounds. Take SGT-3 and SGT6 for example: SGT-3 is composed of Radix Paeoniae alba (12 g, raw) and Radix Glycyrrhizae (8 g, raw), and SGT-6 is composed Radix Paeoniae alba (12 g, raw) and Radix Glycyrrhizae (4 g, raw), the two drugs have the same compatibility of medicines but different dosage. Therefore, the content of chemical compounds is also different. The contents are (mg/mL) 1.59, 0.82, 3.28, 0.486, 3.15, 1.10, 0.27, 0.44, and 0.08 and 1.52, 0.67, 2.91, 0.45, 3.00, 1.07, 0.24, 0.42, and 0.07, respectively.

Different dosage and different compatibility of medicines lead to different content. The possible reasons are that the solubility and chemical properties of the compounds would be changed in virtue of the interactions between herb medicines. Some compounds can affect the PH of the solution and make it acidic or alkaline. Thus, indirectly change the solubility of the other compounds. Sometimes, one compound can react with others and change the chemical properties of the other compounds to form insoluble sediments; these sediments are slowly broken down in the body and gradually play a pharmacodynamics and can make the drug attack slowly, so as to reduce the side effects.

### 4.4. Correlation between Dosage, Compatibility of Medicines, and Therapeutical Effect

We also found that the therapeutical effect of traditional Chinese Medicinal Prescription is influenced by dosage and compatibility of medicines, Different therapeutical effect due to different dosage and different compatibility of medicines.

**Figure 5:**
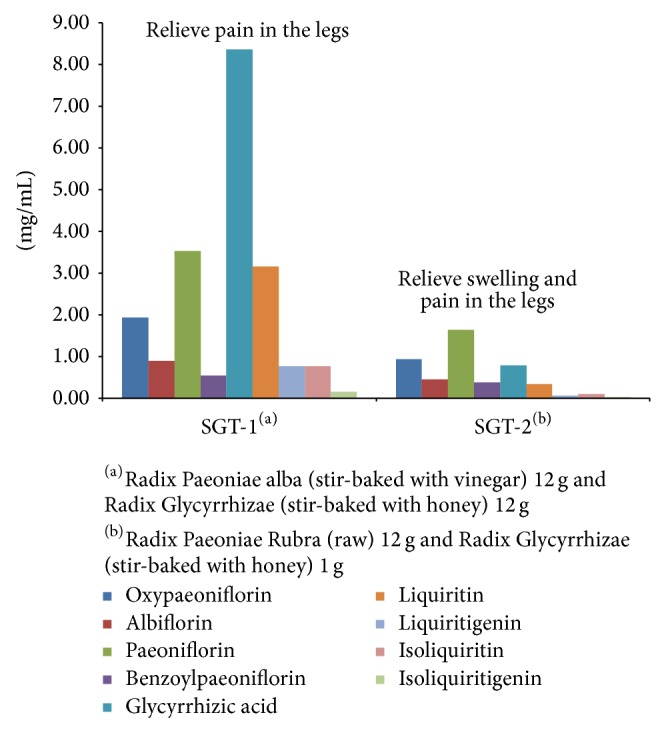
Correlation between therapeutical effects and content of SGT-1 and SGT-2.

**Figure 6:**
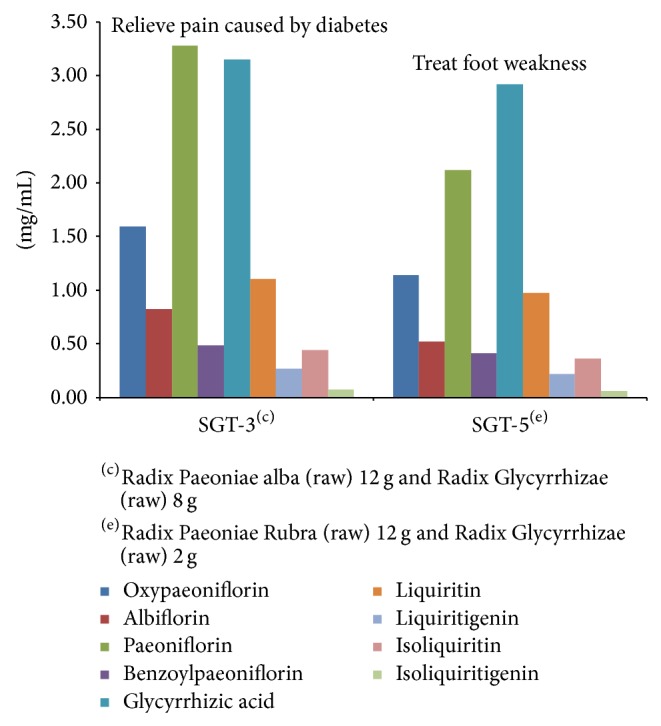
Correlation between therapeutical effects and content of SGT-3 and SGT-5.

**Figure 7:**
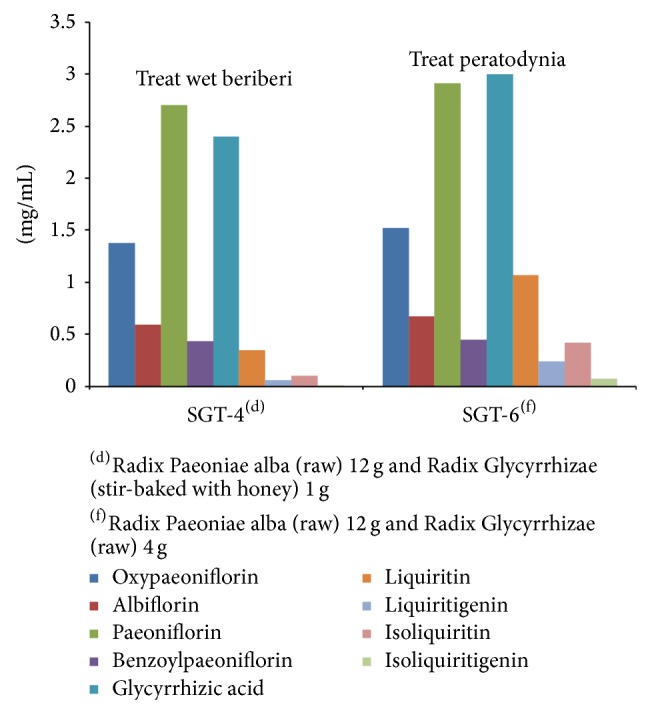
Correlation between therapeutical effects and content of SGT-4 and SGT-6.

One reason is that the contents are different due to different dosage and different compatibility of medicines, which is an important factor in treatment. This conclusion has been already proved and described in the medical classic written by medical expert in ancient china. For example, SGT-1 was used to relieve pain in the legs in* Shang-han-lun* by Zhongjing Zhang in the Han dynasty and SGT-2 was used to relieve swelling and pain in the legs in* Chua-xin-shi-yong-fang* by Yankui Wu in the Song dynasty (Figure 5); SGT-3 was used to relieve pain caused by diabetes and SGT-5 was used to treat foot weakness in* Zhu-shi-ji-yan-fang* by Zhuo Zhu in the Song dynasty (Figure 6); SGT-4 was used to treat wet beriberi in* Wei-shi-jia-cang-fang* by Xian Wei in the Song dynasty and SGT-6 was used to treat peratodyniain* Yi-men-ba-fa* by Hongen Liu in the Qing dynasty (Figure 7).

Another reason is that the compatibility of medicines can play a synergistic effect which can enhance the therapeutical effect. Studies confirmed that glycyrrhizic acid can enhance the therapeutical effect of paeoniflorin on analgesic, antispasmodic, antipyretic, and smooth muscle relaxation.

## 5. Conclusions

The study provides a new HPLC-based method for quality control of the Traditional Chinese Medicinal Prescription SGT. The results of the study showed that SGT mentioned above are different in compatibility of medicines and dosage, and the therapeutic effects of them are different too. The method could be suitable for quality control of SGT with bioactive multicompounds.

## Figures and Tables

**Table 1:** Compatibility of medicines, ratio, and therapeutical effect of SGT in different period.

Reference	Period	Compatibility of medicines	Ratio (w/w)	Therapeutical effect
*Shanghanlun *	In the Han dynasty	Radix Paeoniae alba (Stir-baked with vinegar) and Radix Glycyrrhizae (stir-baked with honey)	1 : 1	For relieving pain in the legs
*Chuanxinshiyongfang *	In the Song dynasty	Radix Paeoniae Rubra (raw) and Radix Glycyrrhizae (stir-baked with honey)	12 : 1	For relieving swelling and pain in the legs
*Zhushijiyanfang *	In the Song dynasty	Radix Paeoniae alba (raw) and Radix Glycyrrhizae (raw)	3 : 2	For relieving pain caused by diabetes
*Zhushijiyanfang *	In the Song dynasty	Radix Paeoniae Rubra (raw) and Radix Glycyrrhizae (raw)	6 : 1	For foot weakness
*Weishijiacangfang *	In the Song dynasty	Radix Paeoniae alba (raw) and Radix Glycyrrhizae (stir-baked with honey)	12 : 1	For wet beriberi
*Yimenbafa *	In the Qing dynasty	Radix Paeoniae alba (raw) and Radix Glycyrrhizae (raw)	3 : 1	For peratodynia

**Table 2:** The binary gradient elution system.

Time (min)	0~5	5~12	12~15	15~20	20~25	25~30	30~40	40~50	50~60

A: acetonitrile (%)	5~10	10~14	14~16	16~18	18~20	20~22	22~25	25~40	40~55

**Table 3:** Wavelengths.

Wavelengths (nm)	230	276	360
Compounds	Oxypaeoniflorin albiflorin, paeoniflorin, benzoylpaeoniflorin, and glycyrrhizic acid	Liquiritin and liquiritigenin	Isoliquiritin and isoliquiritigenin

**Table 4:** Compatibility of medicines and dosage of SGT.

Compatibility of medicines	Dosage	Water
Radix Paeoniae alba (stir-baked with vinegar) and Radix Glycyrrhizae (stir-baked with honey)	12 g and 12 g	240 mL
Radix Paeoniae Rubra (raw) and Radix Glycyrrhizae (stir-baked with honey)	12 g and 1 g	130 mL
Radix Paeoniae alba (raw) and Radix Glycyrrhizae (raw)	12 g and 8 g	200 mL
Radix Paeoniae Rubra (raw) and Radix Glycyrrhizae (raw)	12 g and 2 g	140 mL
Radix Paeoniae alba (raw) and Radix Glycyrrhizae (stir-baked with honey)	12 g and 1 g	130 mL
Radix Paeoniae alba (raw) and Radix Glycyrrhizae (raw)	12 g and 4 g	160 mL

**Table 5:** Linear regression equation, linear range, and limit of detections (LODs) for nine standard substances.

Standard substance	Linear regression equation *y* = *ax* + *b* ^(a)^	Correlation coefficient (*r* ^2^)	Linear range (*μ*g/mL)	LOD (ng/mL)	LOQ (ng/mL)
Oxypaeoniflorin	*y* = 283.4*x* + 2.053	0.9995	0.84~8.40	3.02	9.09
Albiflorin	*y* = 741.8*x* − 13.62	0.9997	1.40~14.00	10.32	30.86
Paeoniflorin	*y* = 800.5*x* + 122.5	0.9995	3.82~38.20	8.34	26.02
Benzoylpaeoniflorin	*y* = 1531*x* − 51.41	0.9997	0.94~9.40	6.23	19.31
Glycyrrhizic acid	*y* = 376.4*x* + 22.81	0.9996	8.80~88.00	16.44	50.64
Liquiritin	*y* = 1072*x* − 73.08	0.9998	2.04~20.40	7.56	22.53
Liquiritigenin	*y* = 4281*x* + 27.72	0.9995	0.90~9.00	8.24	25.54
Isoliquiritin	*y* = 4025*x* + 167.9	0.9997	0.08~0.80	20.11	63.15
Isoliquiritigenin	*y* = 23005*x* + 15.01	0.9998	0.82~8.20	6.89	20.60

^(a)^ *y* and *x* stand for the peak area and the injection quantity (*μ*g) of each standard substance.

**Table 6:** Precision, repeatability, stability, and recovery of nine standard substances.

Standard Substance	Precision	Repeatability	Stability	Recoveries (%)^(a)^	RSD
	RSD (%)	RSD (%)	RSD (%)	(%)	(%)
	(*n* = 6)	(*n* = 6)	(*n* = 5)	(*n* = 9)	
Oxypaeoniflorin	1.65%	1.00%	1.96%	99.00%	1.28%
Albiflorin	1.75%	1.83%	1.19%	99.00%	1.32%
Paeoniflorin	0.58%	1.84%	1.32%	99.00%	2.12%
Benzoylpaeoniflorin	1.91%	2.00%	1.91%	100.00%	2.40%
Glycyrrhizic acid	1.64%	1.75%	1.53%	100.00%	1.18%
Liquiritin	1.20%	1.56%	1.17%	101.00%	2.33%
Liquiritigenin	1.02%	0.92%	1.43%	99.00%	0.89%
Isoliquiritin	1.40%	1.34%	1.43%	100.00%	0.53%
Isoliquiritigenin	1.28%	0.96%	1.29%	101.00%	2.50%

^(a)^Recovery (%) = 100 ∗ (amount found − original amount)/amount spiked.

**Table 7:** The contents of nine compounds in SGT (mg/mL) (*n* = 3).

Compounds	SGT-1^(a)^	SGT-2^(b)^	SGT-3^(c)^	SGT-4^(d)^	SGT-5^(e)^	SGT-6^(f)^
Oxypaeoniflorin	1.92	0.92	1.59	1.38	1.14	1.52
Albiflorin	0.90	0.45	0.82	0.59	0.52	0.67
Paeoniflorin	3.51	1.62	3.28	2.70	2.12	2.91
Benzoylpaeoniflorin	0.53	0.37	0.47	0.43	0.41	0.45
Glycyrrhizic acid	8.35	0.78	3.15	2.40	2.92	3.00
Liquiritin	3.16	0.34	1.10	0.35	0.97	1.07
Liquiritigenin	0.77	0.06	0.27	0.06	0.22	0.24
Isoliquiritin	0.77	0.10	0.44	0.10	0.36	0.42
Isoliquiritigenin	0.15	0.01	0.08	0.01	0.06	0.07

Total content of nine compounds	20.06	4.65	11.20	8.02	8.72	10.35
